# Case Report of Thymoma Tumor Reduction Following Plasmapheresis

**DOI:** 10.1097/MD.0000000000002173

**Published:** 2015-10-30

**Authors:** Wei Jiang, Qitao Yu

**Affiliations:** From the Department of Medical Oncology, Tumor Hospital Affiliated to Guangxi Medical University, Nanning, China.

## Abstract

For thymoma, multidisciplinary antitumor strategy is composed of surgery, chemotherapy, and radiotherapy. Meanwhile, ∼20% to 25% of patients with thymoma have myasthenia gravis and plasmapheresis is recommended for thymoma-associated myasthenia gravis.

We report a case that a 40-year-old woman with thymoma experiencing tumor relapse after surgery showed significant response to plasmapheresis.

This is the first case of thymoma responded to plasmapheresis, which may guide the study of the etiology and pathogenesis of thymoma.

## INTRODUCTION

Thymoma is a common primary tumor in the mediastinum, which originates in the thymus. According to the World Health Organization (WHO) histological classification, thymoma can be categorized into 5 subtypes. Most reports indicate that type A, AB, and B1 were associated with less aggressive clinical behavior and better prognosis than type B2, B3, and thymic carcinomas. However, it is still controversial. The Masaoka system is widely accepted to stage the disease. Treatment strategies of thymoma depend on the stage of disease and include a combination of surgery, chemotherapy, and radiotherapy. As we known, there is no evidence to show definite antitumor activity of other modality in thymoma.

Approximately 20% to 25% of patients with thymoma have myasthenia gravis. The severity of myasthenia gravis is evaluated according to the Medical Scientific Advisory Board of the Myasthenia Gravis Foundation of America clinical classification system (MGFA). For thymoma-associated myasthenia gravis, plasmapheresis is recommended.

We describe here a case of thymoma with myasthenia gravis illustrating significant response to plasmapheresis.

### Case Presentation

A 40-year-old Chinese woman was admitted to our hospital in June 2008 for drooping right eyelid. The patient had been healthy all of her life. A chest computed tomography (CT) scan demonstrated a mediastinal mass. A median sternotomy for a radical thymectomy was performed, and the pathological diagnosis revealed thymoma, histologically classified as WHO type B1. With tumor invading into surrounding fatty tissue, she was indicated as ∏B by the Masaoka staging system. We strongly recommended radiotherapy and chemotherapy after surgery but the patient refused any adjuvant therapy.

Unfortunately, assessed by a routine follow-up CT scan in January 2011, tumor recurred with left pleura dissemination. Positron emission tomography-CT (PET-CT) scan also demonstrated the recurrent lesion. Biopsy via video-assisted thoracic surgery (VATS) confirmed thymoma relapse (type B1, partial B2). Considered unresectable, she was given 3 cycles of chemotherapy consisting of cyclophosphamide, pirarubicin, and cisplatin from February to March 2011. For slight tumor progression, 2 cycles of pemetrexed and Nedaplatin were administered from April to May 2011, and the patient refused further chemotherapy, with stable disease for 30 months.

**FIGURE 1:**
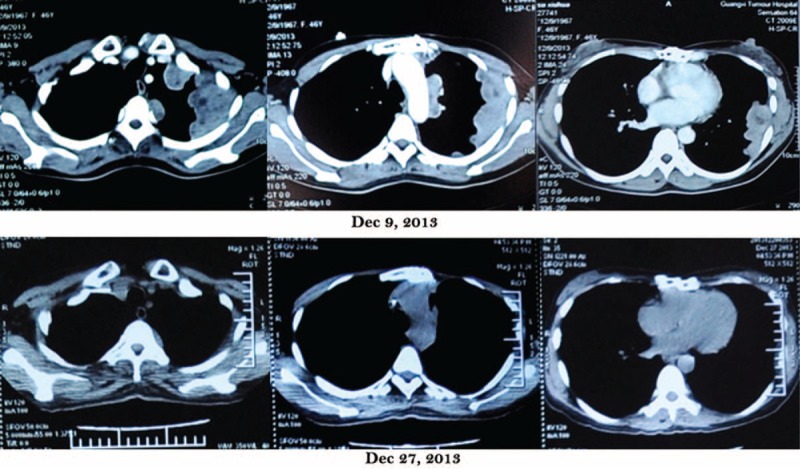
Comparison of CT scan between December 9, 2013, and December 27, 2013. A partial response of tumor following 3 sessions of plasmapheresis was indicated from CT scan. CT = computed tomography.

In 9 December 2013, a follow-up CT scan showed a significant progression of disease. Then, the patient experienced myasthenia gravis with the development of severe acute respiratory failure. She was classified as MGFA class IV. Endotracheal intubation was administrated for ventilator support immediately. In 12, 14, and 16 December, 3 sessions of therapeutic plasma exchanges were performed for blood purification, in which 2000 mL plasma was separated from the blood, discarded in total, and replaced with 2000 mL plasma collected from healthy donors in each session. Each session was followed by perfusion of human immunoglobulins. To our surprise, after therapeutic plasma exchange, not only myodynamia recovered and mechanical ventilation was terminated, but also a partial response of tumor was indicated from CT scan (27 December 2013) although contrasted-enhanced CT was not chosen for a not very well performance status of patient (Fig. 1). She slowly improved to MGFA minimal manifestation status (MMS).

**FIGURE 2:**
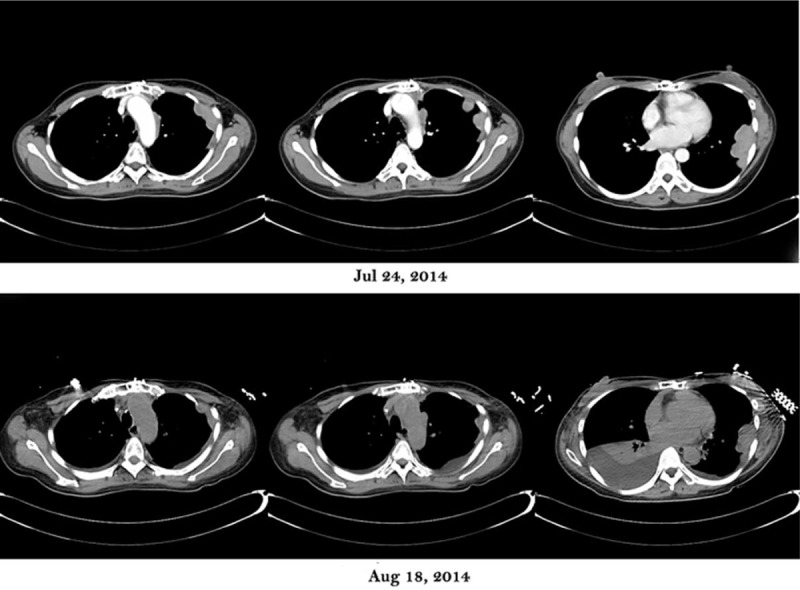
Comparison of CT scan between July 24, 2014, and August 18, 2014. A significant response of tumor following 3 sessions of plasmapheresis was indicated from CT scan. CT = computed tomography.

In July 2014, after 7 months normal living, the patient was referred to us for anhelation. The CT scan showed tumor progression. She quickly progressed to dyspnea and encounter myasthenic crisis again. Given ventilation, the patient received plasmapheresis for 3 sessions in 28, 30 July, and 1 August 2014. The volume exchange of each session is 3000 mL, 2500 mL, and 2000 mL, respectively. Unfortunately, although the lesions showed a decrease in size in CT scan of 18 August 2014 (Fig. 2) and myodynamia recovered, the patient failed of weaning for serious pulmonary infection and passed away in September 2014.

## DISCUSSION

For resectable thymoma, surgery is the mainstay of treatment.^1,2^ This patient had received excision, but recurrence occurred after 32 months. For advanced patients, chemotherapy with or without radiotherapy is recommended.^3,4^ Then, the patient was given chemotherapy but had no chance of radiotherapy, with disease control for >2 years. The survival time of this patient after diagnosis of thymoma was 5 years, which was not comparable of around 90% of the 5-year survival rate for type B1 thymoma in previous reports.^5,6^ Partial type B2 may attribute to a poor prognosis.

However, the most crucial factor shorten the survival time of this patient is serious pulmonary infection associating with myasthenia gravis. Approximately 20% to 25% of patients with thymoma have myasthenia gravis.^7^ Thymoma-associated myasthenia gravis (T-MG) is identified as a tumor originating from thymic epithelial cells, most being cortical subtype (WHO type B).^8^ This patient was classified as type B1, partial B2. She started from drooping right eyelid and showed dyspnea when the tumor significantly progressed, that were both suggestive of myasthenia gravis. For myasthenia gravis, plasmapheresis is a therapeutic modality well established with a strong recommendation.^9^ However, there is no evidence of antitumor activity for this therapeutic method.

We report this case showing significant shrinkage of tumor following plasmapheresis for twice. Until now, the etiological factors that contribute to the development of thymomas remain unknown and the causal mechanism that drives thymoma progression is not clear. We assume that some kind of plasma substances may promote tumor develop in thymoma in this patient. The plasma substances likely had a trophic effect on this patient's thymoma deposits. Recently, a report was published describing an association between myasthenia gravis-associated thymoma and T cell immunoglobulin and mucin domain-3 (Tim-3), which is a subtype of the Tim protein family.^10^ Plasma exchange generally performed to remove pathogenic autoantibodies, immune complexes, cryoglobulins, and toxins that have accumulated in the plasma,^11^ which may eliminate the components stimulating tumor grow and show antitumor efficacy.

## CONCLUSION

To the best of our knowledge, this is the first report that a patient with thymoma responded to plasmapheresis, which suggests that the etiology and pathogenesis of thymoma and the role of plasmapheresis in the patients with T-MG is more complicated than we thought and need further investigation.

### CONSENT

Written informed consent was obtained from the husband of the patient for publication of this case report and any accompanying images. A copy of the written consent is available for review by the Editor of this journal.
